# Dual regulatory effects of gut microbiota and their metabolites in rheumatoid arthritis: balancing pathogenic and protective mechanisms

**DOI:** 10.3389/fimmu.2025.1584023

**Published:** 2025-04-30

**Authors:** Xingwen Xie, Xin Chen, Xuetao Wang, Sunli Wang, Peng Qi

**Affiliations:** ^1^ Affiliated Hospital of Gansu University of Traditional Chinese Medicine, Lanzhou, China; ^2^ Gansu University of Traditional Chinese Medicine, Lanzhou, China

**Keywords:** gut microbiota, intestinal metabolites, gut-joint axis, rheumatoid arthritis, bidirectional regulation

## Abstract

Rheumatoid arthritis is a chronic autoimmune disorder characterized by destructive, symmetric joint inflammation and synovitis, resulting in substantial disability that profoundly compromises patients’ quality of life. Its pathogenesis encompasses complex interactions between genetic and environmental factors. Recent advances in bacterial DNA sequencing technologies have uncovered a significant correlation between the human gut microbiota composition and rheumatoid arthritis progression. Growing clinical and experimental evidence establishes the gut-joint axis as a crucial mediator in rheumatoid arthritis pathogenesis. Comprehensive investigation of gut microbial communities and their metabolites’ influence on rheumatoid arthritis mechanisms, coupled with the elucidation of microbiome’s bidirectional regulatory effects in disease development, not only deepens our understanding of pathological processes but also establishes a theoretical framework for developing novel diagnostic biomarkers and personalized therapeutic interventions to enhance patient outcomes.

## 1 Introduction

Rheumatoid arthritis (RA) is a chronic inflammatory autoimmune disease characterized primarily by polyarticular synovitis, leading to bone and cartilage erosion and ultimately resulting in joint deformity and functional impairment (1). This condition affects approximately 1% of the global adult population (2). While the precise etiology of RA remains elusive, evidence suggests that its pathogenesis involves complex interactions between genetic and environmental factors, triggering aberrant T-cell immune responses and subsequent progressive inflammatory reactions in synovial joints (3–5).

The human microbiome constitutes a sophisticated ecological system essential for health maintenance and disease progression (6–10). The gastrointestinal tract is the most densely populated microbial habitat in humans, with the gut microbiota comprising approximately 70% of the total microbial load (11). The oral cavity contains the second largest microbial community, representing approximately 10% of the total. This oral microbiota plays a crucial role in maintaining oral health. The remaining microbial populations are distributed across other niches, including the skin, respiratory tract, and urogenital tract, where their combined abundance is lower, contributing to the overall diversity of the human microbiome. The human gut microbiome is predominantly composed of phyla including *Firmicutes, Bacteroidetes, Actinobacteria*, and *Proteobacteria* (12), orchestrating essential physiological processes including nutrient metabolism, toxin degradation, immune regulation, and intestinal barrier maintenance (13). Evidence indicates that gut microbiota critically modulate host immune responses, with their dysbiosis strongly correlating with various autoimmune disorders’ pathogenesis (14). In dysbiotic conditions, metabolites—including short-chain fatty acids, polyphenols, vitamins, and tryptophan—produced by proliferating pathogenic strains potentially contribute to RA pathogenesis (15). Moreover, alterations in gut microbiota composition and their metabolic products influence both RA treatment efficacy and drug-induced adverse effects. Thus, elucidating the gut microbiota-RA relationship has profound implications for understanding disease mechanisms, enhancing diagnostic approaches, and developing optimal therapeutic strategies.

## 2 Gut microbiome ecology and its interplay with rheumatoid arthritis pathogenesis

Clinical and experimental studies have established a significant role for the gut-joint axis in the pathogenesis of rheumatoid arthritis (RA) (16, 17). Specifically, *Collinsella* spp. are significantly elevated in patients with stage 1 RA, while *Faecalibacterium* is associated with stage 3 RA, and *Bifidobacterium longum* and *Eggerthella* are enriched in stage 4 RA patients (18). Characterization of the gut microbiome in Chinese RA cohorts has revealed a signature marked by the enrichment of *Lactobacillus salivarius* and a reduction in *Haemophilus* spp (19). Furthermore, meta-analysis has demonstrated a significant alteration in the gut microbial composition of RA patients compared to healthy controls (16). Microbial community diversity is commonly assessed using α-diversity (species richness within a single sample) and β-diversity (differences in microbial composition between samples). Analyses of microbial diversity in RA patients generally indicate reduced or unchanged α-diversity, while β-diversity analyses reveal significant shifts in microbial community structure (20, 21). At the phylum level, the gut microbiota of RA patients is characterized by a significant increase in *Proteobacteria* and *Verrucomicrobia*, and a decrease in *Firmicutes*. Although the Firmicutes/Bacteroidetes (F/B) ratio has not consistently reached statistical significance, its imbalance has been implicated in activating pro-inflammatory pathways by disrupting intestinal barrier integrity (22). At the genus level, RA patients exhibit a significant increase in *Klebsiella*, *Escherichia*, *Eisenbergiella*, and *Flavonifractor*, alongside a significant decrease in *Clostridium*, *Megamonas*, and *Enterococcus* (21, 23–26). Specifically, *Prevotella* spp. have been identified as key pathobionts in RA, with significant enrichment observed in patient fecal samples (21, 27). A 27 kDa protein-derived peptide produced by *Prevotella* can mediate Th1 cell immune responses in early RA patients via HLA-DR (28, 29). Gut microbes and their metabolites exert a dual regulatory role in RA. Metabolomic analyses have revealed elevated levels of glycerophospholipids, benzene and its derivatives, and cholesterol in RA patients, coupled with decreased levels of sphingolipids and tryptophan-derived downstream metabolites (21). Furthermore, fecal butyrate levels are significantly reduced in RA patients. Butyrate, known for its anti-inflammatory properties, can inhibit arthritis progression, a finding supported by animal studies showing that exogenous butyrate supplementation suppresses arthritis (21). The reduced levels of tryptophan-derived downstream metabolites in RA patients, along with the correlation between serum tryptophan and its metabolites with disease activity and rheumatoid factor, suggest that gut microbes and their metabolites influence RA development. Systematically elucidating the molecular mechanisms underlying the gut microbiome’s association with RA pathogenesis will provide critical insights for understanding disease mechanisms, developing diagnostic markers, and optimizing therapeutic strategies.

## 3 Mechanistic insights into gut microbiome and metabolite-driven pathogenesis of rheumatoid arthritis

**Figure 1:**
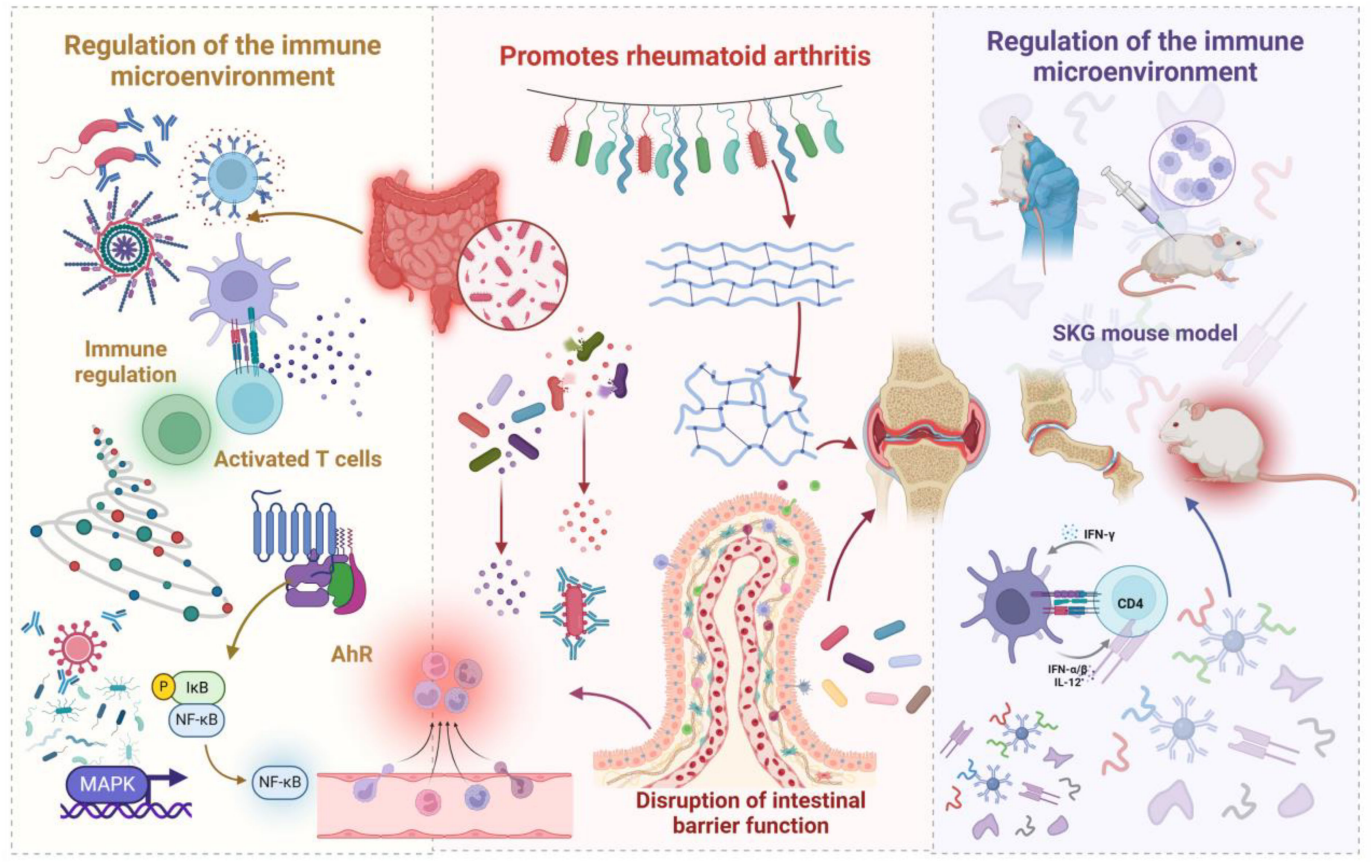
Schematic illustration depicting how gut microbiota and their metabolites promote the development of RA. Dysbiosis of the gut microbiome leads to an increase in harmful bacteria, which, by modulating the immune microenvironment, triggers inflammation. This microbial imbalance also enhances intestinal permeability, facilitating bacterial translocation that activates immune responses and initiates a cascade of events leading to the destruction of bone and cartilage. The immune cross-reactivity mechanism, supported by molecular simulations, elucidates how microbial antigens, due to their structural similarity to self-proteins, induce autoimmunity, subsequently causing inflammatory damage to joint tissues.

Gut microbiota dysbiosis is increasingly recognized as a pivotal factor in the pathogenesis of RA, primarily by modulating intestinal barrier integrity and immune system function (30, 31). Immune dysregulation resulting from microbial imbalance affects both innate and adaptive immunity (16). Within innate immunity, a disrupted microbiota can lead to aberrant activation of pattern recognition receptors, subsequently inducing the upregulation of pro-inflammatory cytokines and the downregulation of anti-inflammatory mediators, thereby disrupting local immune homeostasis. In adaptive immunity, a dysbiotic microbiota mediates the initiation and perpetuation of autoimmune responses by modulating antigen-presenting cell function, influencing T cell subset differentiation, and regulating B cell activation states. Microbiota-driven inflammation can compromise the tight junctions between intestinal epithelial cells, leading to increased intestinal permeability (32). However, while increased epithelial barrier permeability is a prerequisite for substance translocation, it is not the sole determinant. Although heightened permeability allows for greater translocation of microbes, their metabolites, and antigenic components into the systemic circulation, the specific substances critically involved in RA pathogenesis depend on the precise composition of the barrier-crossing metabolites and bacterial constituents. Specific bacterial components, such as lipopolysaccharide, can elicit significant systemic inflammation due to their potent immunostimulatory properties, even in small quantities. Similarly, imbalances in the levels of specific microbial metabolites, such as short-chain fatty acids (SCFAs), can contribute to the RA pathological process by influencing immune cell function (33). Therefore, while increased intestinal barrier permeability facilitates the entry of microbes and their metabolites into the host, the etiology of RA is contingent upon the specific types and concentrations of metabolites and bacterial components that traverse the barrier (34). Further elucidation of RA pathogenesis requires a deeper understanding of the precise mechanisms underlying increased intestinal barrier permeability, the identification of key metabolites and bacterial components that effectively translocate across the epithelial barrier, and the assessment of their relative importance in RA development. It can be hypothesized that increased intestinal barrier permeability, the composition of specific metabolites, and the presence of particular bacterial components act synergistically to mediate the translocation of substances across the epithelial barrier (35), ultimately influencing systemic immune responses and promoting the onset and progression of RA. **Figure 1** illustrates the proposed role of gut microbes and their metabolites in promoting rheumatoid arthritis development.

### 3.1 Immunological microenvironment modulation

The gut microbiota orchestrates RA pathogenesis through complex immunological networks. In innate immunity, dynamic signaling between gut-associated lymphoid tissue immune cells and intestinal microbiota establishes critical immune barriers (36). Microbial dysbiosis initiates aberrant innate immune cell activation, elevating pro-inflammatory cytokines (Interleukin-6 (IL-6), tumor necrosis factor - alpha (TNF-α), Interleukin-beta (IL-1β), Interleukin-12, Interleukin-23 (IL-23)) while suppressing anti-inflammatory mediators (transforming growth factor beta (TGF-β), Interleukin-10 (IL-10)) (31, 37). The gastrointestinal tract, as the body’s largest immune organ, engages in bidirectional communication with the host immune system to precisely balance immune homeostasis and inflammatory responses (38).

In adaptive immunity, microbiota primarily modulates the T helper 17/regulatory T cells (Th17/Treg) axis. After microbial antigen presentation by dendritic cells and macrophages to CD4+ T cells, these lymphocytes differentiate into distinct functional subsets (39, 40). RA patients display elevated peripheral Th17 cells, secreting pro-inflammatory cytokines (Interleukin-17 (IL-17), Interleukin-21, Interleukin-22 (IL-22), granulocyte-macrophage colony-stimulating factor, TNF-α) that induce synovial fibroblast receptor activator of nuclear factor kappa-B ligand (RANKL) expression. RANKL/osteoprote gerin imbalance drives osteoclastogenesis and bone erosion while promoting pannus formation and synovial angiogenesis (41, 42). Conversely, Treg cells maintain immunological homeostasis by secreting IL-10 and TGF-β1, thereby suppressing Th17 activity (43–45). The Th17/Treg balance regulation exhibits strain-specific microbial control. Clinical investigations reveal inverse correlations between *Firmicutes* abundance and Th17 cells in RA patients (46), while *Bacteroides fragilis* colonization enhances immunomodulation through Treg activation (47, 48). *Faecalibacterium prausnitzii* exerts protective effects by maintaining intestinal barrier integrity, modulating the Th17/Treg balance, and suppressing inflammatory responses (49). Studies in germ-free K/BxN mice demonstrate that *Segmented filamentous bacteria* colonization promotes functional Th17 cell differentiation via two mechanisms: elevated ileal serum amyloid A mediating dendritic cell-driven Th17 differentiation, and ATP-activated CD70highCD11clow cells inducing IL-6, IL-23p19, and TGF-β expression through integrin-αV/-β8 signaling (50–52).

Microbiota further modulates T follicular helper and regulatory cell equilibrium. Decreased T follicular regulatory cells correlate with enhanced RA activity and autoantibody production, inversely relating to *Ruminococcus* levels (53). Microbial antigens cooperate with T follicular helper cells to trigger B cell hyperactivation, enhancing pathogenic autoantibody production (18). Bacterial peptidoglycan detection in RA synovium (29, 54) confirms altered microbiota’s role in immune dysregulation (19, 55, 56). The aryl hydrocarbon receptor (AhR) pathway critically influences RA immunoregulation, particularly in RANKL-mediated osteoclastogenesis through nuclear factor kappa-light-chain-enhancer of activated B cells/mitogen-activated protein kinase (NF-κB/MAPK) signaling (57, 58). AhR additionally regulates immune surveillance via Th17/Treg modulation (59), with pathway disruption promoting autoimmunity and systemic inflammation.

### 3.2 Enhanced intestinal mucosal barrier permeability

The intestinal barrier system consists of multiple structural components: mucus layer, epithelial cell layer, basement membrane, vascular endothelium, and immune molecules. This system maintains precise regulation of innate and adaptive immune responses under physiological conditions, enabling selective barrier function while facilitating nutrient absorption (44). Zonulin, a protein secreted by intestinal epithelial cells, primarily functions to modulate tight junction protein complexes between these cells, thereby influencing intestinal barrier permeability. Increased expression or activity of zonulin can alter the structure and function of tight junction proteins, leading to enlarged intercellular spaces and a consequent increase in intestinal barrier permeability, which allows larger molecules to translocate into the bloodstream via the paracellular pathway. Clinical studies have demonstrated a concurrent increase in serum and fecal zonulin levels, providing evidence for the association between intestinal barrier dysfunction and RA (60). Gut microbiota dysbiosis is a critical factor in inducing intestinal barrier dysfunction. Studies have revealed an abnormal increase in the abundance of Collinsella spp. in RA patients (27, 61), and the molecular mechanisms by which these bacteria disrupt the intestinal barrier include the downregulation of tight junction protein expression in intestinal epithelial cells and the activation of the NF-κB1-mediated inflammatory pathway. Furthermore, metabolites produced by Collinsella, such as α-aminoadipic acid and asparagine, synergize with IL-17A to further exacerbate intestinal barrier damage (62). The regulatory relationship between microbial composition and intestinal permeability has been validated in HLA-DQ8 transgenic mouse models. Experimental evidence indicates that overexpression of Collinsella aerofaciens increases the incidence of collagen-induced arthritis by suppressing the expression of tight junction proteins in intestinal epithelial cells (63). Increased intestinal permeability facilitates the translocation of microbes and their metabolites across the compromised barrier into the systemic circulation, subsequently activating local tissue immune responses (37). This can initiate a cascade of destructive events in bone and cartilage tissues (64–66).

### 3.3 Molecular simulation insights into self-antigen recognition and rheumatoid arthritis pathogenesis

Molecular mimicry constitutes a mechanism wherein structural or sequence homology between pathogenic microorganisms and host molecules generates cross-reactive immune responses that trigger autoimmunity. In RA pathogenesis, this process operates through cross-recognition between microbial antigens and self-protein epitopes. Clinical investigations demonstrate that RA patient T and B cells recognize N-acetylglucosamine-6-sulfatase and filamin A as autoantigens (52% and 56%, respectively) (67) These autoantigens share extensive sequence homology with *Prevotella*-derived bacterial proteins within human leukocyte antigen-DR presentation regions. Through cross-recognition, anti-*P. copri* immune responses activate autoantigen-specific lymphocyte responses, inducing autoantibody production. These autoantibodies display marked RA specificity, occurring significantly less frequently in healthy individuals and patients with other autoimmune conditions (67). The spleen tyrosine kinase deficient mouse model has illuminated molecular mimicry’s role in RA pathogenesis. This model harbors zeta-chain-associated protein kinase 70 gene mutations that impair T cell receptor signaling, potentiating T cell autoreactivity (68). In specific pathogen-free environments, *P. copri* colonization enhances Th17 responses and arthritis manifestations in spleen tyrosine kinase deficient mice. Notably, germ-free mice maintain arthritis resistance despite fungal exposure, underscoring the gut microbiota’s essential role in autoreactive T cell activation (68). Mechanistic studies reveal that *P. copri* monocolonization amplifies immune responses to the arthritis-associated autoantigen 60S ribosomal protein L23a in lymphoid tissues (69). The molecular mimicry-based mechanism of immune cross-reactivity elucidates how microbial antigens, through their structural resemblance to self-proteins, can trigger autoimmune responses, subsequently leading to inflammatory damage in joint tissues. Beyond the immune-inflammatory responses mediated by molecular mimicry, gut microbial metabolites may also play a pathogenic role in RA. Paralleling the mechanism of microbial metabolite-induced bone loss in periodontitis, we hypothesize that gut microbiota dysbiosis in RA patients leads to the aberrant production of specific metabolites, such as SCFAs and lipopolysaccharide, which may translocate to the joints via a compromised intestinal barrier. Locally within the joints, these metabolites may directly activate osteoclasts or indirectly induce synovial cells to release pro-inflammatory cytokines and bone-resorbing factors, thereby promoting the destruction of joint bone. Thus, microbial metabolite-induced bone resorption may constitute another significant pathway through which the gut microbiota influences RA pathological progression, acting in concert with molecular mimicry mechanisms to promote disease onset and development. This finding offers a novel perspective on understanding the pathogenesis of RA.

## 4 Mechanistic insights into gut microbiome and metabolites’ inhibitory effects on rheumatoid arthritis pathogenesis

**Figure 2:**
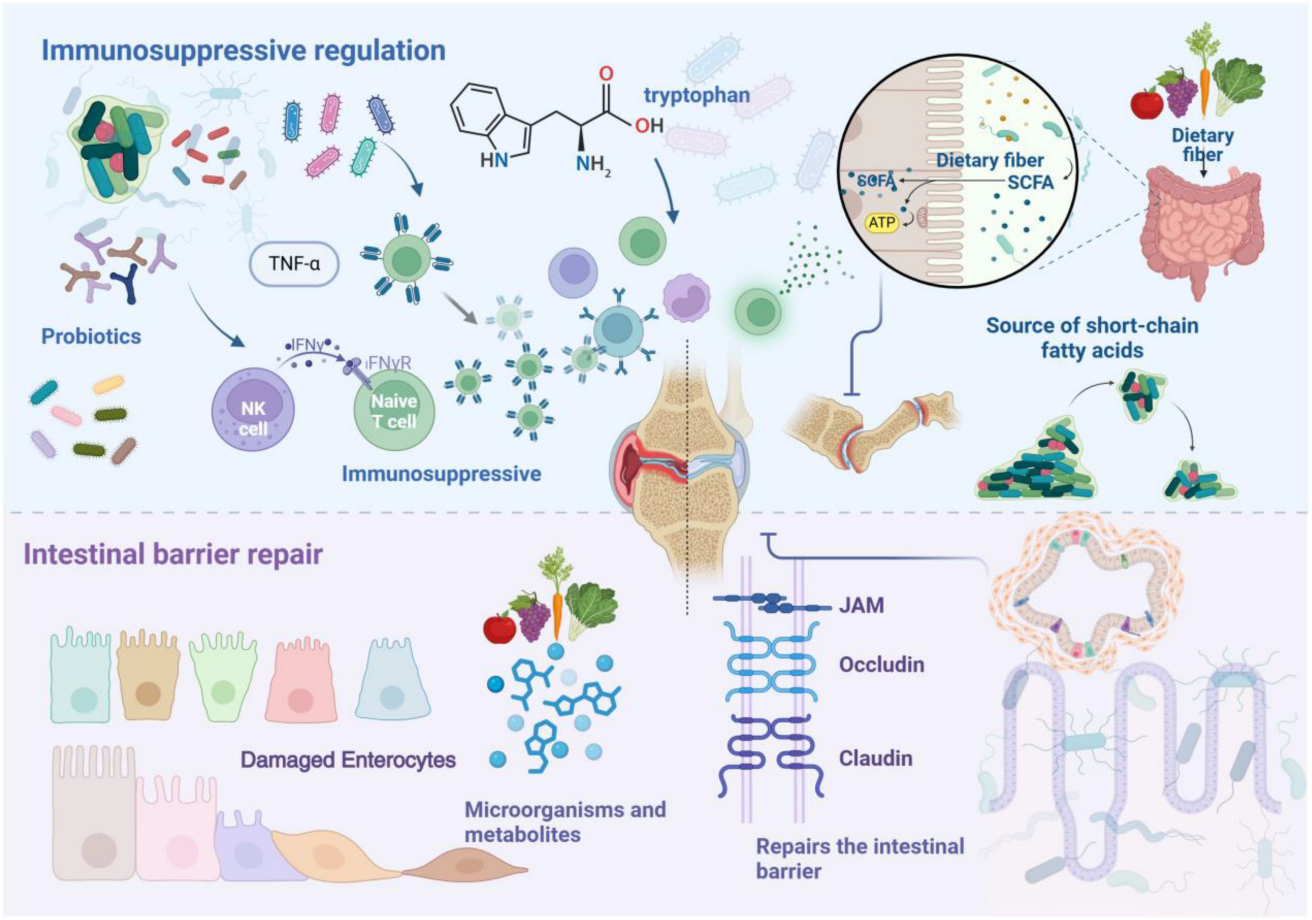
Schematic illustration showing how gut microbiota and their metabolites inhibit the development and progression of RA. Probiotics and their metabolites exert inhibitory effects on RA by modulating immune cell functions and regulating the expression of inflammatory cytokines. Short-chain fatty acids play a key role in this process. Additionally, various nutritional factors contribute to the repair and maintenance of the intestinal barrier by modulating microbiota composition and metabolic pathways. Dietary fiber, an essential nutrient for maintaining barrier integrity, when adequately consumed, reduces serum levels of Zonulin and calprotectin, thereby inhibiting the onset and progression of RA.

Gut microbiota dysbiosis exerts bidirectional effects on RA pathogenesis. Beneficial microbes maintain intestinal homeostasis through immune modulation and barrier preservation, particularly *Lactobacillus*, *Bifidobacterium*, and *Bacteroides fragilis* species, which attenuate RA manifestation and progression (70, 71). Clinical studies have characterized distinct alterations in RA patients’ gut microbiota, revealing depletion of multiple genera including *Bacteroides*, *Streptococcus*, *Rhodococcus*, *Prevotella*, *Haemophilus*, and *Parabacteroides* (72–74). These compositional shifts influence host immunity through altered metabolic profiles. The tryptophan metabolic pathway emerges as a critical mediator of RA suppression, where microbe-derived tryptophan metabolites function as aryl hydrocarbon receptor ligands to regulate both innate and adaptive immunity (75). Metabolomic profiling has identified reduced concentrations of tryptophan-derived compounds in RA patients’ fecal samples, specifically N-methyl-5-hydroxytryptamine, 5-hydroxyindoleacetic acid, kynurenic acid, xanthurenic acid, and 3-hydroxyanthranilic acid, underscoring tryptophan metabolism’s pivotal role in RA pathogenesis (21). **Figure 2** illustrates how gut microbiota and their metabolites inhibit the development and progression of RA.

### 4.1 Immunosuppressive regulatory mechanisms

Gut microbiota and their metabolites attenuate RA progression through modulation of immune responses and inflammatory pathways. Probiotic strains exhibit distinct immunosuppressive properties, with *Lactobacillus casei*, *L. rhamnosus*, and *Lactococcus lactis* demonstrating therapeutic potential (76, 77). Mechanistic studies suggest that *Lactobacillus casei* may induce anti-inflammatory responses by influencing the maturation and function of dendritic cells, upregulating the expression of the anti-inflammatory cytokine IL-10, and downregulating the levels of pro-inflammatory cytokines IL-6 and TNF-α, thereby leading to reduced C-reactive protein levels and improved arthritis symptoms. *Lactobacillus rhamnosus*, on the other hand, primarily mediates immunosuppressive effects by modulating the expression of the pro-inflammatory cytokine IL-1 (27). Despite elevated detection in severe RA patients (78, 79), *L. salivarius* exhibits anti-inflammatory activity in collagen-induced arthritis models, mitigating bone erosion and neutrophil infiltration. *Falcatibacterium prausnitzii* reduces pro-inflammatory cytokines IL-17, IL-1β, TNF-α while enhancing commensal microbiota proliferation, particularly *Akkermansia* and *Bilophila* (27).

SCFAs and bile acid metabolites serve as key immunomodulators. In arthritis models, butyrate drives follicular regulatory T cell development, attenuating autoimmune progression (80). Propionate may influence the balance between Th17 cells and regulatory T cells. Metabolites produced by *Parabacteroides distasonis*, including lithocholic acid, deoxycholic acid, isoallolithocholic acid, and 3-oxolithocholic acid, exert anti-arthritic effects through synergistic action. Specifically, 3-oxolithocholic acid and isoallolithocholic acid act as TGR5 receptor agonists, inducing macrophage polarization towards the M2 phenotype and inhibiting Th17 cell differentiation (81). The tryptophan metabolic pathway emerges as a critical immunoregulator in RA, with clinical studies revealing diminished tryptophan metabolites in RA synovial fluid versus osteoarthritis samples (82). The serotonin metabolite 5-hydroxyindoleacetic acid promotes regulatory T cell development via AhR activation (83). Microbial SCFAs enhance regulatory B cell function through elevated 5-hydroxyindoleacetic acid levels, reducing arthritis manifestations (84). Additionally, kynurenic acid limits synovial proliferation (21), while 3-hydroxyanthranilic acid attenuates lipopolysaccharide-induced macrophage inflammation through NF-κB inhibition (85). Dietary composition significantly influences the composition of the gut microbiota and the production of its metabolites, thereby indirectly modulating these immunosuppressive mechanisms. Signaling molecules generated by the gut microbiota and their metabolites can traverse the bloodstream to impact the systemic immune system, including immune responses in distal sites such as the joints.

### 4.2 Intestinal mucosal barrier restoration

The intestinal barrier functions as a crucial regulator of organismal homeostasis, with its impairment contributing to autoimmune disease pathogenesis. Diverse nutritional factors orchestrate barrier maintenance and repair by modulating microbial ecosystems and metabolic networks. Dietary fiber constitutes an essential nutritional element in preserving barrier integrity, reducing serum concentrations of both the permeability regulator Zonulin and the inflammatory biomarker calprotectin (86). Furthermore, dietary fiber maintains mucus layer stability, thereby influencing microbial community structure (87). SCFAs fortify intestinal barrier function while serving as microbial energy substrates and community regulators. These metabolites enhance barrier integrity through three primary mechanisms: tight junction protein induction, epithelial cell regeneration, and antimicrobial peptide production. Butyrate demonstrates particular efficacy in promoting tight junction protein expression (88). Vitamins contribute to barrier homeostasis through epithelial cell regulation: vitamin D coordinates tight junction assembly and cell survival (88), while vitamin E strengthens barrier function by supporting butyrate-producing bacteria and maintaining microbial homeostasis (89). Select amino acids emerge as critical mediators of barrier function. Glutamine and tryptophan insufficiency directly compromises barrier integrity, elevating intestinal permeability (90, 91). Plant polyphenols quercetin, myricetin, kaempferol, and curcumin reinforce barrier function by augmenting transepithelial electrical resistance and enhancing tight junction protein expression zonula occludens-1 and claudin-1 (92), consequently attenuating RA progression.

## 5 Translational applications of gut microbiome in rheumatoid arthritis diagnosis and treatment

**Figure 3:**
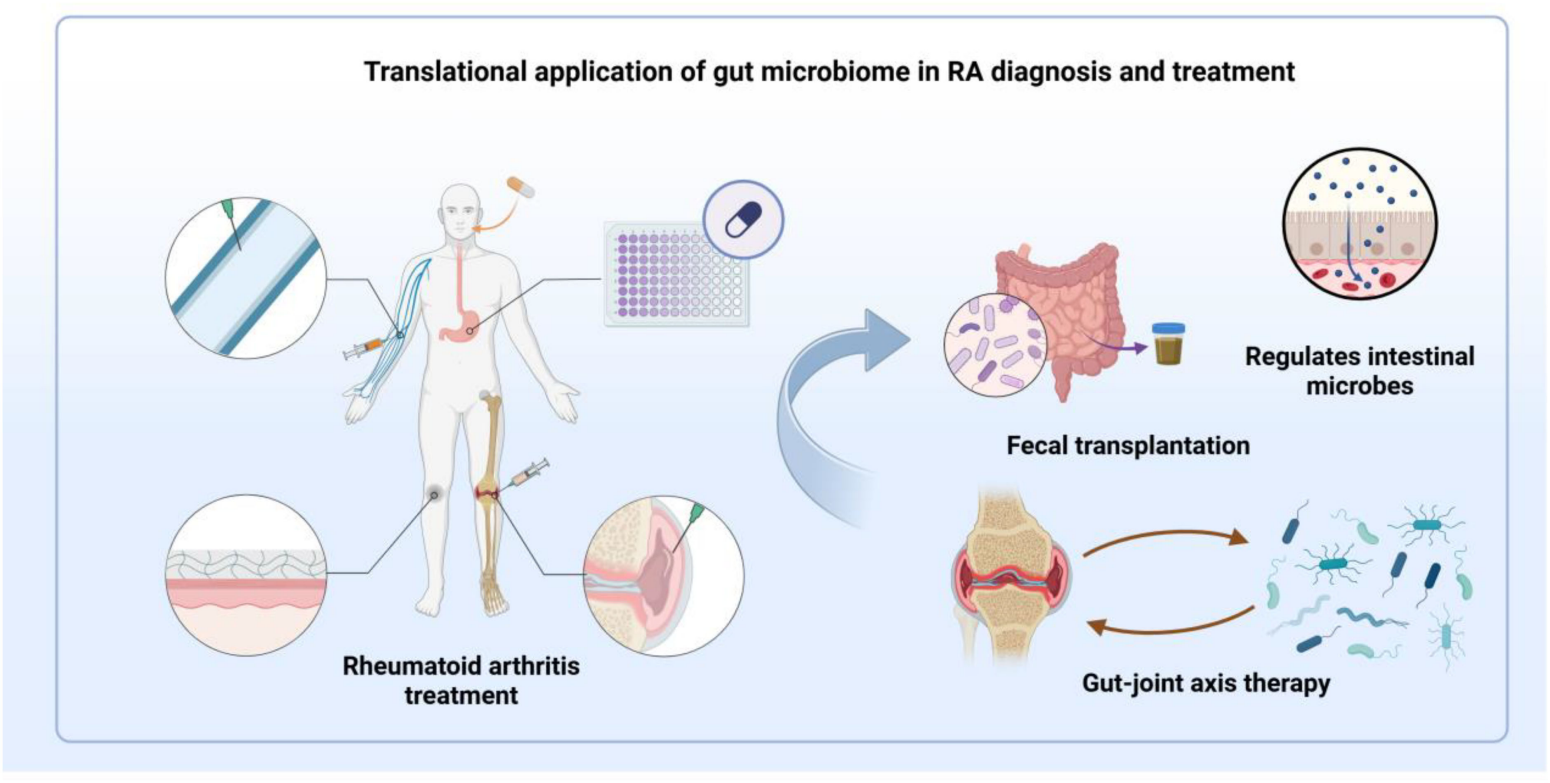
Schematic illustration of the translational applications of the gut microbiome in the diagnosis and treatment of RA. While RA can be managed through various methods, including oral medications, clinical outcomes exhibit significant individual variability. Interventions such as probiotic supplementation and stabilization of the intestinal barrier show potential therapeutic benefits in RA. By precisely modulating microbiota composition, interventions like probiotics, fecal microbiota transplantation, and therapies targeting the gut-joint axis may pave the way for the development of novel diagnostic biomarkers and personalized treatment strategies.

Dysregulation of gut microbial composition and diversity drives RA pathogenesis and progression through immune system modulation (65). Therapeutic strategies targeting intestinal microbiota represent an emerging frontier in RA treatment. Methotrexate, while established as the primary RA therapeutic, exhibits marked inter-patient variability in clinical outcomes. Such heterogeneity encompasses both pharmacodynamic responses sensitivity and tolerability and pharmacokinetic parameters distribution and clearance across renal, hepatic, and serosal compartments. Gut microbiota and their enzymatic products modulate drug efficacy through multiple mechanisms, influencing the bioavailability, therapeutic response, and adverse effects of various medications, particularly methotrexate. Reciprocally, these therapeutics and their metabolites reshape microbial communities, thereby altering immune function. Interventions targeting intestinal homeostasis have demonstrated promising clinical outcomes in RA management, with preclinical studies validating the therapeutic potential of microbiome modulation and barrier function enhancement (93). Furthermore, leveraging immune reactivity against selected gut bacterial species holds potential clinical utility. Exploring the detection of specific antibodies or T cell responses against particular gut bacteria in RA patients could lead to the development of novel diagnostic biomarkers, enabling earlier or more precise RA diagnosis. Moreover, immune reactivity against these specific gut bacteria may also offer new insights into therapeutic strategies (94). Developing specific immunotherapies, such as vaccines or antibodies, targeting pathogenic bacteria to inhibit their growth or mitigate their induced inflammatory responses warrants consideration. Inducing immune tolerance to specific gut bacteria could also be a strategy to alleviate inflammation driven by these bacteria. These advances suggest that targeted manipulation of microbial ecosystems may yield novel diagnostic markers and personalized interventions. **Figure 3** shows the translational applications of the gut microbiome in the diagnosis and treatment of RA. This microbiome-centric approach establishes new paradigms for precision medicine in RA treatment.

### 5.1 Biomarker discovery and diagnostic tool development

The composition of the gut microbiota and serum cytokine profiles are emerging as valuable molecular biomarkers for the early diagnosis of rheumatoid arthritis (RA), monitoring disease activity, and evaluating treatment responses. Microbiome sequencing analyses have revealed characteristic alterations in the gut microbiota of RA patients, including an increased ratio of Bacteroidetes to Firmicutes (24, 95). Community structure analysis further indicates that early RA patients exhibit an increased abundance of *Prevotella* and a decreased abundance of *Bacteroides*, a pattern with potential diagnostic utility. The pathogenic role of the microbiota in RA development has been validated in germ-free SKG mouse transplantation models. Patients with active disease display specific microbial signatures, such as reduced levels of *Haemophilus* and increased *Streptococcus salivarius*. Compared to patients in remission, those with active disease show a significant increase in the relative abundance of *Collinsella* and *Akkermansia*, suggesting that these microbial shifts may serve as effective indicators for monitoring disease activity (3, 96).

RA-associated autoantibodies, such as rheumatoid factor, anti-citrullinated peptide antibodies, and anti-carbamylated antibodies, can be detected in serum years before the clinical onset of RA (97–99). Although established RA typically involves the joints, the disease’s pathogenesis may originate beyond the articular structures, potentially linking to citrullination in the lungs or periodontal tissues, the gut microbiota, or adipose tissue. The normal appearance of synovial tissue in anti-citrullinated peptide antibodies-positive individuals with arthralgia supports this notion (100). The oral microbiome in patients with rheumatoid arthritis exhibits significant dysbiosis, which can be restored with RA treatment, and the extent of this restoration correlates with the patient’s response to therapy (101). Epidemiological studies have shown a high prevalence of periodontitis in RA patients. Periodontitis, a chronic inflammatory disease of the oral cavity, suggests a close relationship between the oral microbiome and RA. The periodontal anaerobe *Porphyromonas gingivalis* can synthesize bacterial peptidylarginine deiminase involved in RA pathogenesis (102). *P. gingivalis* is currently the only known bacterium that expresses peptidylarginine deiminase and is significantly associated with RA. Continuous peptidylarginine deiminase secretion by *P. gingivalis* leads to the citrullination of α- and β-fibrin chains in the joint synovium, generating autoantigens that drive anti-citrullinated peptide antibodies production. anti-citrullinated peptide antibodies then forms immune complexes with citrullinated proteins, binding to immune-inflammatory cells via Fc and C5a receptors, triggering a complex cascade of reactions and the release of inflammatory mediators, ultimately leading to local synovial inflammation and the development of RA (103).

Analysis of serum cytokine profiles provides crucial insights for evaluating disease activity and treatment responses. Elevated levels of pro-inflammatory cytokines TNF-α and IL-6 reflect increased disease activity, while decreased levels of the anti-inflammatory cytokine IL-10 suggest impaired immunosuppressive function. Integrating the characteristic changes in microbiota composition and cytokine profiles can facilitate the construction of a multi-dimensional diagnostic system based on the gut-joint axis (3, 96). Next-generation high-throughput sequencing technologies and metabolomics analysis platforms have enhanced the precision of identifying gut microbiota and their metabolites. The application of multi-omics data combined with machine learning algorithms offers robust technical support for developing more accurate and sensitive diagnostic tools for RA. Machine learning algorithms can integrate gut microbiota data, cytokine profiles, and other clinical data to improve the accuracy and sensitivity of RA diagnosis. Furthermore, multi-omics data can be used to identify different subtypes of RA patients, enabling personalized treatment strategies. Next-generation high-throughput sequencing technologies allow for the precise analysis of gut microbiota composition and diversity, while metabolomics analysis platforms provide a more comprehensive analysis of gut metabolites, aiding in the discovery of novel biomarkers.

### 5.2 Microbiome modulatory mechanisms in existing therapeutic paradigms

Disease-modifying antirheumatic drugs modulate RA progression through restructuring intestinal microbial communities. Clinical studies reveal elevated relative expression units of *Bacteroides* and *Prevotella* species in disease-modifying antirheumatic drugs-treated patients’ fecal samples, concurrent with decreased *Clostridium* abundance (25). Antibiotic intervention studies establish the microbiome’s causative role in RA pathogenesis: four-week clindamycin treatment selectively depletes anaerobic bacteria, intensifying both incidence and severity of collagen-induced arthritis in murine models (104).

Natural compounds demonstrate therapeutic efficacy through regulation of microbial ecosystems and metabolic networks. Berberine administration specifically reduces *Prevotella* while enriching butyrate-producing bacteria, attenuating collagen-induced arthritis symptoms via enhanced butyrate synthesis, intestinal hypoxia stabilization, and nitrate metabolism modulation (105). The parasitic immunomodulator excretory/secretory products of *Acanthocheilonema viteae*, derived from *Acanthocheilonema*, preserves microbiota homeostasis and barrier function when administered subcutaneously, preventing arthritic manifestations in collagen-induced arthritis models (106). *Tripterygium wilfordii* extract restores microbial equilibrium by suppressing pro-inflammatory taxa (*Desulfovibrio*, *Mucor*, *Helicobacter*, Spirochaetaceae), thereby attenuating tissue damage and inflammatory cytokine production such as TNF-α, IL-1β, IL-6, IL-7, IL-23 (88).

Paeoniae radix glycosides normalize gut microbial taxonomy in collagen-induced arthritis models, enhancing commensal populations while suppressing inflammatory mediators, including secretory Immunoglobulin A and Interferon gamma (107). Microbial metabolites emerge as therapeutic targets, with SCFAs mitigating RA progression through concurrent inhibition of T helper 1 differentiation and promotion of Treg proliferation (108). Clematis saponins regulate SCFAs homeostasis, with linear discriminant analysis effect size validating their role in maintaining Gram-negative/positive bacterial balance (109), consequently ameliorating RA symptoms.

### 5.3 Microbiome-mediated mechanisms of therapeutic efficacy modulation

The gut microbiota emerges as a potential therapeutic target in RA, with targeted modulation strategies encompassing probiotic interventions, prebiotic supplementation, and fecal microbiota transplantation. These microbiome-based therapeutic approaches ameliorate disease progression through restoration of intestinal microbial homeostasis. Such microbiota-mediated interventions, operating through multiple regulatory layers, establish novel paradigms for precision RA treatment.

#### 5.3.1 Therapeutic strategies modulated by microbiome regulation

Probiotics demonstrate therapeutic potential through diverse mechanisms. They suppress inflammatory signaling pathways by downregulating Toll-like receptor expression. These pattern recognition receptors mediate immune cell activation and inflammatory mediator production through pathogen-associated molecular pattern recognition. Probiotics additionally induce immunoregulat forkhead box P3 ory factor secretion by antigen-presenting cells. Experimental studies confirm their promotion of forkhead box P3-positive regulatory T cell differentiation, thereby inhibiting inflammatory cascades. Furthermore, *Lactobacillus* and *Bifidobacterium* species maintain intestinal barrier integrity through short-chain fatty acid production (71). Clinical investigations validate probiotic therapeutic efficacy. RA patients in remission (DMARD-naïve) demonstrate significantly improved health scores following 12-month *L. rhamnosus* intervention (110). Combined supplementation with *L. acidophilus*, *L. casei*, and *B. bifidum* reduces disease activity scores and high-sensitivity C-reactive protein levels (111, 112). Multi-strain formulations, including *LC-11, LA-14, LL-23*, *BL-04*, and *BB-06*, exhibit significant efficacy in active RA patients (113).

Microbial metabolites play pivotal roles in maintaining intestinal barrier function. Butyrate and short-chain fatty acids ameliorate inflammation associated with leaky gut syndrome by enhancing tight junction protein expression (114, 115). Lithocholic acid demonstrates anti-inflammatory activity in collagen-induced arthritis models (116, 117), while *Bacteroides fragilis* and propionate serve as adjuvant therapeutic factors, enhancing conventional treatment efficacy (118). Indole-3-methanol inhibits adjuvant-induced arthritis progression (119). Sinomenine attenuates RA-associated inflammation by elevating microbiota-derived indolic tryptophan metabolites, activating AhR, and modulating NF-κB/MAPK signaling pathways (16).

#### 5.3.2 Microecosystem reconstruction

Fecal Microbiota Transplantation (FMT) constitutes a therapeutic approach that rehabilitates recipient microbial ecosystems via healthy donor microbiota transfer. Mechanistic studies reveal that normal microbiota transplantation enhances bone mass regeneration through orchestration of microbial metabolism and immune networks (120). The interplay between dysbiosis and mucosal immunity emerges as a critical initiating factor in RA pathogenesis (121). Investigations using germ-free BALB/c ZAP-70W163C mutant models demonstrate that RA patient-derived FMT intensifies arthritic manifestations via Th17-mediated signaling cascades (122). FMT, which involves the transfer of gut microbiota from a healthy donor, aims to reconstruct the recipient’s intestinal microecosystem. Its mechanisms of action extend beyond the direct supplementation of beneficial bacteria to encompass complex modulation of the host immune system. FMT reduces systemic inflammation by regulating the production of gut metabolites, influencing host immune cell function, and repairing the compromised intestinal barrier, thereby limiting the translocation of bacteria and their metabolites into the systemic circulation. Furthermore, FMT can modulate the balance of immune cells within the intestine of RA patients, increasing the number of Tregs and decreasing the proportion of pro-inflammatory Th17 cells. With the continuous advancement of FMT techniques, its application in clinical translational research for RA is gaining momentum (123). Preliminary clinical studies have observed some symptom relief in a subset of RA patients; however, larger-scale clinical trials are needed to validate its efficacy due to the limited sample sizes of current studies. The efficacy of FMT is influenced by multiple factors, including donor selection, microbial composition of the transplant, and the route of administration (124). Therefore, precise analysis of the gut microbiota composition of healthy donors and optimization of delivery methods (e.g., oral capsules, enemas) are crucial for enhancing the clinical efficacy of FMT. Nevertheless, FMT carries potential risks of infection, particularly for immunocompromised RA patients. The long-term effects of microbiota transplantation on the host remain largely unknown and necessitate long-term follow-up studies. Further refinement is required regarding the optimization of FMT administration routes, long-term efficacy, and safety assessments. Systemic FMT treatment holds promise for providing a novel therapeutic target for the precision treatment of RA by reshaping the diversity and functional network of the gut microbiota in affected individuals. This strategy underscores a new research direction for RA treatment by modulating RA-related immune responses from the perspective of restoring microecological balance.

### 5.4 Translational clinical strategies for microbiome-based therapeutics

Triptolide orchestrates anti-arthritic effects by modulating microbial communities and metabolic networks. Mechanistic investigations demonstrate its therapeutic action through three primary pathways: enhancement of *Lactobacillus* populations, upregulation of microbiota-derived tryptophan metabolites, and AhR signaling activation for inflammatory suppression (81). Moreover, triptolide-enriched *Paracasei* and *L. casei* communities mitigate RA manifestations through potentiated tryptophan metabolism and AhR pathway stimulation. Photobiomodulation therapy attenuates TNF-mediated RA progression via gut-joint axis modulation (125). This intervention reconstructs microbial ecosystems, facilitating beneficial bacterial expansion - particularly *Clostridium*, *Lactobacillus*, and *Escherichia* species - thus reinforcing symbiotic network integrity. Microbial amino acid metabolites emerge as key mediators in photobiomodulation’s regulation of the gut-joint axis, with efficacy determined by precise intervention protocols, metabolomic signatures, and tissue-specific enzymatic activities (125). Although non-steroidal anti-inflammatory drugs suppress RA symptoms, they commonly disrupt intestinal mucosal integrity and microbial homeostasis (74). Evidence indicates that probiotic supplementation counteracts non-steroidal anti-inflammatory drugs-induced enteropathy, with *Bifidobacterium*, *Lactobacillus*, and *Prevotella* species demonstrating robust mucosal protection in preclinical models (126), underscoring the microbiome’s therapeutic potential.

## 6 Conclusion

Gut microbiota and their metabolites orchestrate critical regulatory networks in RA pathogenesis. RA patients demonstrate enrichment of *Klebsiella*, *Escherichia*, *Eisenbergiella*, and *Flavobacterium*, concurrent with depletion of beneficial microorganisms, particularly *Bifidobacterium*, *Streptococcus*, and SCFA-producers. Pathogenic bacteria accelerate RA progression through inflammatory cascade activation and barrier compromise, while beneficial microbes attenuate disease development via anti-inflammatory mechanisms and barrier reinforcement. The microbiome emerges as a promising therapeutic and diagnostic frontier, with specific microbial populations representing potential intervention targets.

Therapeutic strategies aimed at modulating the gut microbiome and restoring intestinal microecological balance have demonstrated the potential to regulate the immune system, offering new avenues for the treatment of RA. However, several limitations remain. Current research is largely correlational, observing alterations in gut microbial composition and metabolite levels in RA patients. Whether these changes are a direct cause or a consequence of RA requires further investigation. Furthermore, the gut microbiota and metabolite profiles of RA patients exhibit significant inter-individual variability, potentially influenced by genetic, environmental, and lifestyle factors. The application of gut microbiota modulation strategies in RA treatment is still in its early stages, necessitating more extensive clinical trials to validate their efficacy and safety. The development of diagnostic and therapeutic tools for RA based on the gut microbiota and its metabolites also warrants further research. Future endeavors should prioritize more clinical studies to verify the effects and mechanisms of gut homeostasis regulation in RA prevention and treatment. Exploring the mechanisms of specific probiotics or metabolites, developing novel therapies targeting the gut microbiome, and integrating these approaches with traditional pharmacological treatments and lifestyle interventions will offer more comprehensive strategies for RA management. With a deeper understanding of the intricate relationship between the gut microbiota and its metabolites and the immune system, future research should emphasize the integrated analysis of multi-omics data, the combined use of animal models and clinical trials, and the development of personalized treatment strategies. Notably, exploring the interplay between the gut microbiota and drug metabolism in RA patients, and investigating the therapeutic potential of modulating gut microbes and their metabolites holds promise for novel insights and therapeutic targets for the clinical prevention and treatment of RA, ultimately leading to improved patient outcomes and enhanced quality of life.
